# Spatial encoding in spinal sensorimotor circuits differs in different wild type mice strains

**DOI:** 10.1186/1471-2202-9-45

**Published:** 2008-05-21

**Authors:** Jonas Thelin, Jens Schouenborg

**Affiliations:** 1 Neuronano Research Center, Department of Experimental Medical Science, Lund University, BMC F10, S-221 84, Sweden

## Abstract

**Background:**

Previous studies in the rat have shown that the spatial organisation of the receptive fields of nociceptive withdrawal reflex (NWR) system are functionally adapted through experience dependent mechanisms, termed somatosensory imprinting, during postnatal development. Here we wanted to clarify 1) if mice exhibit a similar spatial encoding of sensory input to NWR as previously found in the rat and 2) if mice strains with a poor learning capacity in various behavioural tests, associated with deficient long term potention, also exhibit poor adaptation of NWR.

The organisation of the NWR system in two adult wild type mouse strains with normal long term potentiation (LTP) in hippocampus and two adult wild type mouse strains exhibiting deficiencies in corresponding LTP were used and compared to previous results in the rat. Receptive fields of reflexes in single hindlimb muscles were mapped with CO_2 _laser heat pulses.

**Results:**

While the spatial organisation of the nociceptive receptive fields in mice with normal LTP were very similar to those in rats, the LTP impaired strains exhibited receptive fields of NWRs with aberrant sensitivity distributions. However, no difference was found in NWR thresholds or onset C-fibre latencies suggesting that the mechanisms determining general reflex sensitivity and somatosensory imprinting are different.

**Conclusion:**

Our results thus confirm that sensory encoding in mice and rat NWR is similar, provided that mice strains with a good learning capability are studied and raise the possibility that LTP like mechanisms are involved in somatosensory imprinting.

## Background

Understanding how sensory information is encoded in spinal sensorimotor circuits and adapted to the body anatomy and biomechanical properties during development are key issues in neuroscience. A system suitable to investigate this issue is the nociceptive withdrawal reflex system (NWR). In the adult rat, the NWR has a modular organisation; each module controls a single muscle and performs a detailed sensorimotor transformation [1,2]. The cutaneous receptive field of each module has a sensitivity distribution that is an imprint of the withdrawal efficacy of the muscle itself. In other words, the strength of the connections between cutaneous afferent fibres and neurones in the central reflex network is proportional to the withdrawal action of the output muscle [3]. Within nucleus proprius in the lower lumbar cord, narrow rostrocaudally extended zones receive a convergent cutaneous input that is weighted in same way as in individual NWR modules. The sensory input to these areas thus appear to be encoded in a motor frame of reference ("action based") rather than being a direct map of the body [4]. Hence, by mapping the receptive fields of hindlimb NWRs, it is possible to assess the sensory encoding in the lower lumbar cord.

Several findings in the rat provide evidence that the somatosensory imprint on the reflex modules is the result of an experience dependent tuning during development [1]: 1) An extensive reorganisation of the receptive fields for NWRs occurs over the first 3 postnatal weeks [5], 2) The reflex modules adapt to both altered innervation and to new movement patterns due to a tendon transfer, if these interventions are made at birth [6], 3) the adaptation is blocked by local anaesthesia [7], 4) tactile feedback on spontaneous muscle twitches guide the NWR network adaptation [8]. The latter findings indicate the presence of cross-modality mechanisms in somatosensory imprinting. In addition, these findings demonstrated for the first time that spontaneous movements reflect ongoing learning in somatosensory circuits whereby they acquire information about the body anatomy and movement patterns. Since somatosensory imprinting has a fundamental role in adapting the connections of nervous system to the body anatomy and biomechanics it is of considerable interest to clarify the underlying molecular mechanisms. Given that the varieties of genetically modified mice available provide useful tools to analyse these mechanisms, we wanted to clarify whether spatial encoding of sensory input to mice NWRs abide the same principles as previously found in rats. An additional aim of the present study was to assess if mice strains with poor learning capabilities in various behavioural tasks, also exhibit poor adaptation of the NWRs. To these ends, we used four different "wild-type" strains of mice, two of which (DBA/2 and 129S6/SvEvTac) exhibit poor results in behavioural tasks (spatial learning) [9,10]and impaired LTP in the hippocampus [11]. The other two strains (NMRI and C57BL/6) used in this study have a normal LTP in hippocampus and a normal spatial learning [12,13].

## Methods

### Animals used

We used four different strains of mice (NMRI n = 6, C57BL/6 n = 6, 129S6/SvEvTac n = 6 and DBA/2 n = 6) in the NWR mapping. All mice were bought from Taconic *M&B *A/S, (P.O. box 1079, DK-8680 Ry, Denmark).

The animals received food and water ad libitum and were kept in a 12-h day-night cycle at a constant environmental temperature of 21°C (humidity 65%). Approval for the experiments was obtained in advance from the Malmö/Lund ethical committee on animal experiments.

### Preparation

The animals were anaesthetized with halothane/isoflurane (0.9 – 2.0%), in a mixture of 65% nitrous oxide and 35% oxygen, and were ventilated artificially via a tracheal cannula. The expiratory CO_2 _was monitored continuously. An infusion of 5% glucose in Ringer acetate (pH = 7.4) at a rate of 0.2–0.5 ml/h was administered via the right jugular vein. Mean arterial blood pressure (55 – 140 mmHg) was monitored continuously in the right carotid artery. Core temperature was maintained between 36.5 and 38.5°C using a thermostatically controlled, feedback-regulated heating system. Local infiltration of 2.0 mg/ml lidocaine (Xylocaine) with 1.2 μg/ml adrenaline was used to reduce nociceptive input during surgery. A craniotomy was performed, and the brain rostral to the inferior colliculus was removed. The halothane/isoflurane anaesthesia was then discontinued. A laminectomy of the tenth thoracic vertebra was carried out and the mouse was spinalized at the thoracic segment T 10–11 using a pair of fine scissors. The spinal chock lasted less than 10 minutes in the mice studied. To ensure stable conditions, recordings were commenced at least one hour after the spinalization. The total recording time did not exceed 1 hour. No significant change in reflex amplitude occurred during the recording time.

For EMG recordings, a small opening was made in the skin overlying the muscle belly, and a reference electrode was inserted in an adjacent skin flap. After termination of the experiments, the animals were given a lethal dose of halothane.

### Recording data

A computerized method, termed receptive field imaging (RFI), for rapid mapping of multiple receptive fields and their respective sensitivity distributions was used in all experiments. This method has been fully described and validated in [14]. In brief, stimulation and recording, with spike detection and counting, were performed on-line by this system. All raw data sweeps were stored to permit further off-line analyses. The RFI system allows repeated receptive field measurements in a time range of minutes. Key features of this system are a random stimulation of specific standard sites on the skin and an averaging procedure that calculates the strength of the input from each of the stimulated sites. The sampling frequency was 10 kHz/channel, and a 12-bit voltage resolution of the total assigned voltage span. See below for details on the subsequent imaging of the receptive fields.

### Cutaneous stimulation

A CO_2_-laser was used (Irradia, beam diameter 3 mm, intensity 5 W) to elicit NWRs in hindlimb muscles. This method allows a precise temporal and spatial stimulation selectively of cutaneous nociceptive Aδ and C afferent fibres in the epidermis [14]. 16 standard sites on the ventral side of the hind paw were stimulated in a random order. The threshold was defined as the CO_2_-laser stimulation duration that elicited an EMG response, usually 6–10 ms, in three out of five trials in each animal. Stimulation during recording was usually 4–8 ms above the threshold to ensure a reflex response. This would increase the skin temperature 5–10°C, at a depth of 100 μm, above the nociceptive threshold [15].

### Electromyography recordings

Fine steel needle electrodes, insulated except for about 80 μm at the tip were used for EMG recordings. The recording electrodes were inserted into the mid-region of each muscle belly. The identity of the muscles was determined by observing the movements evoked by cathodal pulses (100 Hz, 200 ms, 20 μA, 20 pulses), delivered via an exploring electrode [16]. Generally, the EMG activity in three hind limb muscles (M. Peroneus longus, M. Tibialis anterior, M. Semitendinosus) was recorded simultaneously in each experiment.

The EMG recordings were amplified; high pass filtered (50 Hz) and monitored both acoustically and on a computer screen. Judging from spike amplitudes, the recordings usually contained multiple motor units. The number of motor unit spikes was counted on-line by computer software and all raw data were stored on hard disc.

### Analysis

Topographical representation of receptive fields: For each muscle in each mouse, total response magnitude on stimulation (counted during 0–900 ms after onset of laser pulse) were normalized and expressed as percentage of the maximal response. A mean of five stimulations was then calculated for each muscle in each mouse. From these mean values, an average receptive field, divided into three areas of differing sensitivity: Maximal sensitivity (70–100% of maximum), medium sensitivity (30–70% of maximum) and low sensitivity (< 30% of maximum), was constructed. The areas of different sensitivity were delimited with the aid of computer generated isoresponse lines (Kriging algorithm and contour program, Surfer 6.01 from Golden software Inc. 809 14^th ^street, Golden, Colorado 80402-1866).

Reflex response latency measurements: Reflex responses usually had latencies > 100 ms, i.e. they were presumably due to C-fibre input and we therefore compared onset latencies in the C-fibre range. Responses with latencies < 100 ms (judged as A-fibre responses) and > 400 ms (activity likely arising from other sources) were not included. The onset latency was defined as the time passed from stimulus onset until the first 2 ms bin containing at least half the number of spikes of the bin with the highest number [17]. This method has previously been found to yield accurate estimates of onset latencies. All histograms were inspected visually and if responses were judged too small to allow reliable latency estimations these values were excluded from the results. When compensating for differences in afferent fibre length, a C-fibre conduction velocity of 0.8 m/s was assumed [18].

### Statistical analysis

In all receptive field mappings, each standard site of stimulation was stimulated five times. The mean response amplitude for each site was then calculated and normalized. When comparing receptive fields we used the mean response amplitude values obtained from corresponding standard sites on the skin. The Spearmans correlation coefficient was then calculated with GraphPad Prism 4.0 and used as a measure of similarity between the NWR receptive fields [14]. The correlations values were calculated from the mean of five consecutive maps in each animal and muscle. The comparison of location of foci of the receptive fields was done with unpaired t-test. All comparisons of C-fibre latency between the strains and variation within the NWR receptive fields were done with one-way ANOVA with Dunnetts post-hoc test. Significance levels are given as * P < 0.05, ** P < 0.01, ***P < 0.001.

## Results

In the first part of this study, the spatial encoding of sensory input to NWR modules (M. peroneus longus, M. Tibialis anterior and M. Semitendinosus) was studied in the NMRI and the C57BL/6 mouse strains (normal LTP) and compared with that previously found in rats. Data in the rat were taken from the work of Petersson and co-workers (2001) n = 10 which used the same mapping techniques as applied here. A total of 15 maps were obtained from each mouse. As in rats, the reflex responses on CO_2 _laser stimulation was dominated by a late reflex discharge with an onset latency of about 150 ± 30 ms and a total response duration of 400–700 ms. Also the reflex threshold was similar to that previously found in rats (8 ± 2 ms CO_2 _duration).

### Spatial organisation of receptive fields

**Figure 1:**
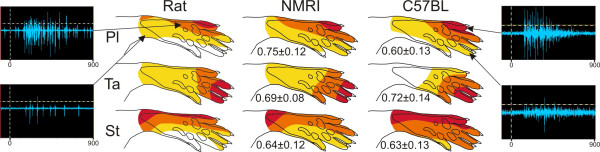
**Comparison of the rat NWRs receptive fields, to NMRI (n = 6) and C57BL (n = 6) mice for three hind limb muscles, peroneus longus (Pl), semitendinosus (St) and tibialis anterior (Ta).** For each muscle, responses on stimulation were normalized and expressed as percentage of the maximal response. The receptive fields were divided into zones of maximal sensitivity (70–100% of maximum), medium sensitivity, (30–70% of maximum) and low sensitivity (< 30% of maximum) and presented in different colours, dark red, maximal sensitivity, yellow, low sensitivity. The mean ± SD of the Spearman correlation for receptive fields are presented. The mean values from five consecutive mappings in each muscle were used for correlation analysis. Raw data EMG recordings from m. peroneus longus are shown on each side. The upper recordings show activity from the stimulated focus area for the respective muscles. The recordings shown in the lower pictures illustrate muscle activity when stimulated peripheral to the focus area for respective muscle. Time points (-100 ms and 900 ms with respect to start of stimulation) are shown below each graph, the horizontal dashed line indicates the threshold for counting spikes and the vertical dashed line shows the time of stimulation onset.

As can be seen in Fig. 1, a strong correlation (range of mean values from each individual: 0.60–0.75) between the response amplitudes obtained on stimulation of different sites within the receptive fields was found for all the muscles examined. These correlations were statistically significant for all the muscles studied (range of P values < 0.05 – 0.001). This finding indicates that the spatial organisation of the receptive fields of NWRs in these two species is organized in the same way. Since the hind limb anatomy and withdrawal movement patterns on contraction in single muscles are very similar for the mouse and the rat, it follows that the sensitivity distribution in NWRs in NMRI mice is also an imprint of the withdrawal movement pattern (see Introduction). We henceforth use the NMRI strain as the normal (with respect to NWR) mice strain when analyzing the NWR receptive fields of other mice strains.

We then compared the sensorimotor transformation in four mice strains, two of which have defects in hippocampal LTP. The 129S6/SvEvTac mice have difficulties to induce and maintain LTP in the hippocampus. The DBA/2 strain exhibit a normal induction but a poor maintenance of LTP. Furthermore, both strains are poor learners in spatial learning task, such as the T-maze [10,11]. The NMRI and C57BL/6 mouse exhibit good results in different learning tasks compared to other mouse strains [13,12]. The spatial organisation of receptive fields, thresholds, onset latency for C-fibre evoked responses, overall reflex gain and response variation were determined in individual reflex modules.

**Figure 2:**
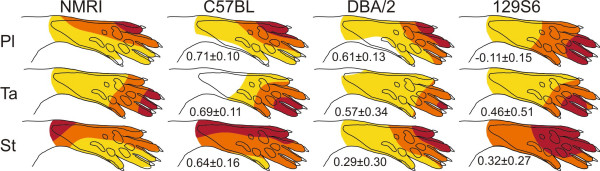
**Comparison of NWRs receptive fields in four different strains of mice in three hind limb muscles, peroneus longus (Pl), semitendinosus (St) and tibialis anterior (Ta).** Each map is a mean of five maps recorded in one mouse. The mean ± SD of Spearman correlation, NMRI (n = 6) compared to C57Bl/6 (n = 6), DBA/2 (n = 6) and 129S6/SvEvTac (129S6) (n = 6) is indicated in each map. The mean values from five consecutive mappings in each muscle were used for correlation analysis. Note that in the 129S6/SvEvTac mice, the sensitivity in the receptive field for ST muscle had a rather flat distribution explaining why only two sensitivity levels (30–70% and 70–100% of maximal response) are indicated in the figure.

Comparing the spatial organisation of the receptive fields of NMRI strain with that in C57Bl/6 mice (normal LTP) we found a relatively high correlation for all muscles studied (range of mean values was 0.64–0.71). These correlations were statistically significant for all the muscles studied (range of P values < 0.05 – 0.001). By contrast, mice strains with deficient LTP; DBA/2 and 129S6/SvEvTac mice, had a more variable correlation coefficients with the NMRI strain and ranged from (range of mean values) -0,11 to 0,61 for the muscles studied (Fig. 2). Lowest correlations were found between NMRI and 129S6/SvEvTac mice strains. Generally, the NWR receptive fields of peroneus longus and semitendinosus were more distorted than that of tibialis anterior. For example, in the 129S6/SvEvTac mice, the peroneus longus receptive field exhibits large aberrant foci on the central digits but not on digit 5 (normal focus). Relatively strong responses were also elicited from the medial side of the plantar skin. Since the peroneus longus move the medial side of the paw towards stimulation, this sensitivity distribution often results in erroneous movements. Similarly, the receptive field of the NWR of the semitendinosus muscle in the 129S6/SvEvTac and DBA/2 was markedly disturbed as compared to the NMRI mice. These differences in location of foci were statistically significant for the peroneus longus and the semitendinosus muscles (p < 0.01–0.001, unpaired t-test, for 129S6/SvEvTac and p < 0.05–0.01 for DBA/2 as compared to NMRI). The receptive field of the NWR of the tibialis anterior muscle in 129S6/SvEvTac and DBA/2 strains exhibited no statistically significant difference in foci compared to the foci in NMRI. It can be concluded that the mice strains with a deficient hippocampal LTP studied here also show a deficient somatosensory imprinting.

### Response thresholds and latencies within receptive field

**Figure 3:**
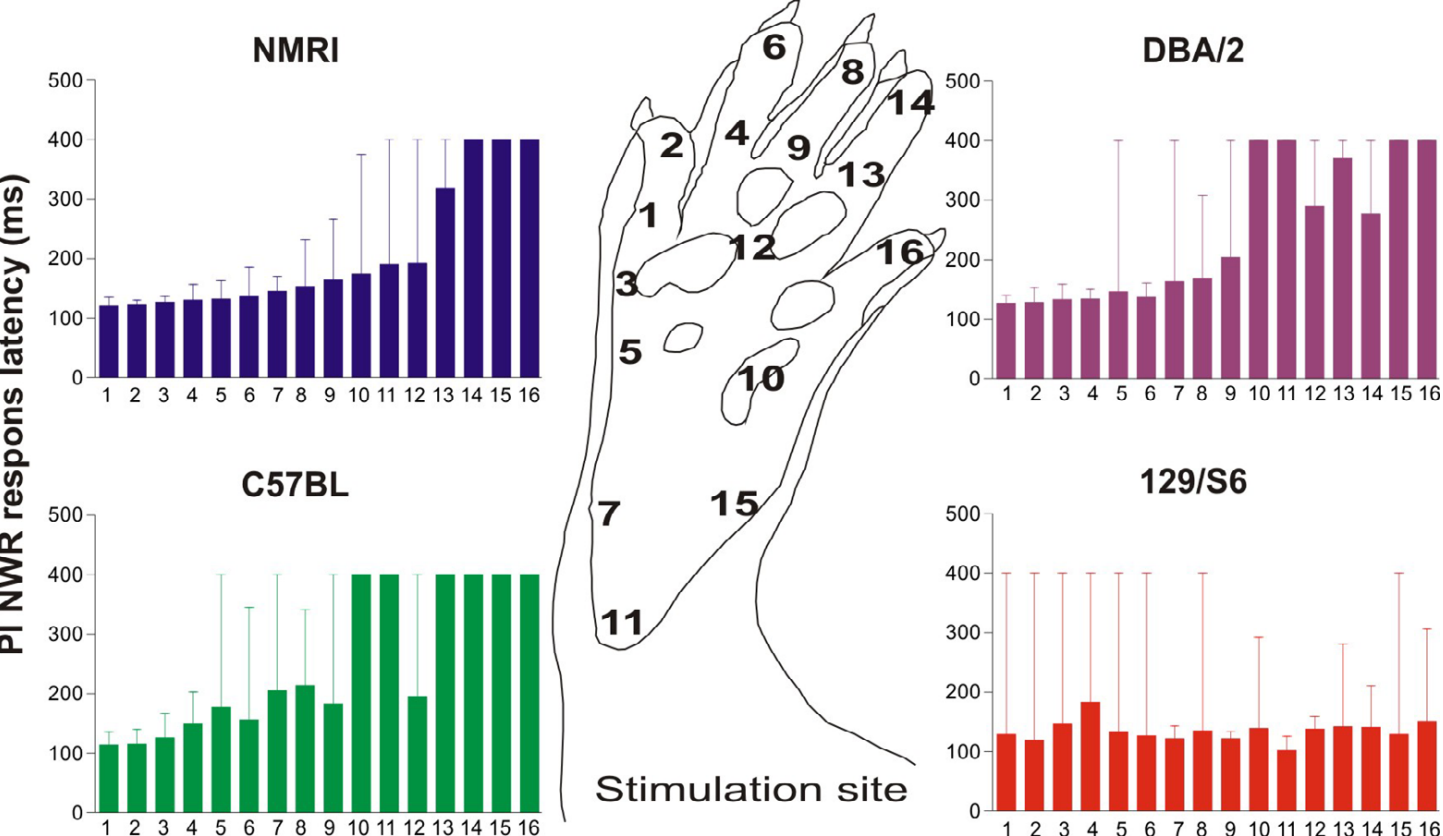
**Onset latency of C-fibre evoked NWR in m. peroneus longus in four different strains of mice (n = 6 from each strain).** The C-fibre latency is presented as the median and the error bars indicate interquartile range. When there was no muscle activity the latency was set to 400 ms. The data are compensated for differences in afferent fibre length. The shortest latency is normally found in the focus of the receptive fields. Note the small differences in latency between stimulation sites in the 129S6/SvEvTac strain as compared to the other mouse strains.

To clarify if the deficient somatosensory imprinting is accompanied by a general change in sensitivity of NWRs, the thresholds and latencies of reflexes evoked in peroneus longus were analyzed in the four mice strains. No significant differences in NWR thresholds were found (one-way ANOVA), irrespective of whether the thresholds were measured in the normal focus or in the aberrant foci for peroneus longus in respective mouse strain. Likewise, no difference in onset C-fibre latency between respective present focus or "normal" focus (here defined as the focus present in the NMRI mouse strain) on digit 5, was found between the different mice strains (one-way ANOVA). (Fig. 3). The shortest onset C-fibre latency is normally found in the focus of the receptive fields. To assess differences in latency between different sites within the receptive fields all the C-fibre latency data were compensated for differences in afferent fibre length (to spinal cord) with a C-fibre conduction velocity of 0.8 m/s [18]. In Figure 3 it can be seen that NMRI, C57BL, DBA/2 exhibited increased response latencies toward the periphery of their receptive fields. By contrast, the 129S6/SvEvTac strain exhibited only small differences in C-fibre latency between stimulation sites as compared to the other strains studied. This "flat" distribution of onset latencies in 129S6/SvEvTac mice presumably reflect a more even sensitivity distribution within their receptive fields than in the other strains.

### Response variation

**Figure 4:**
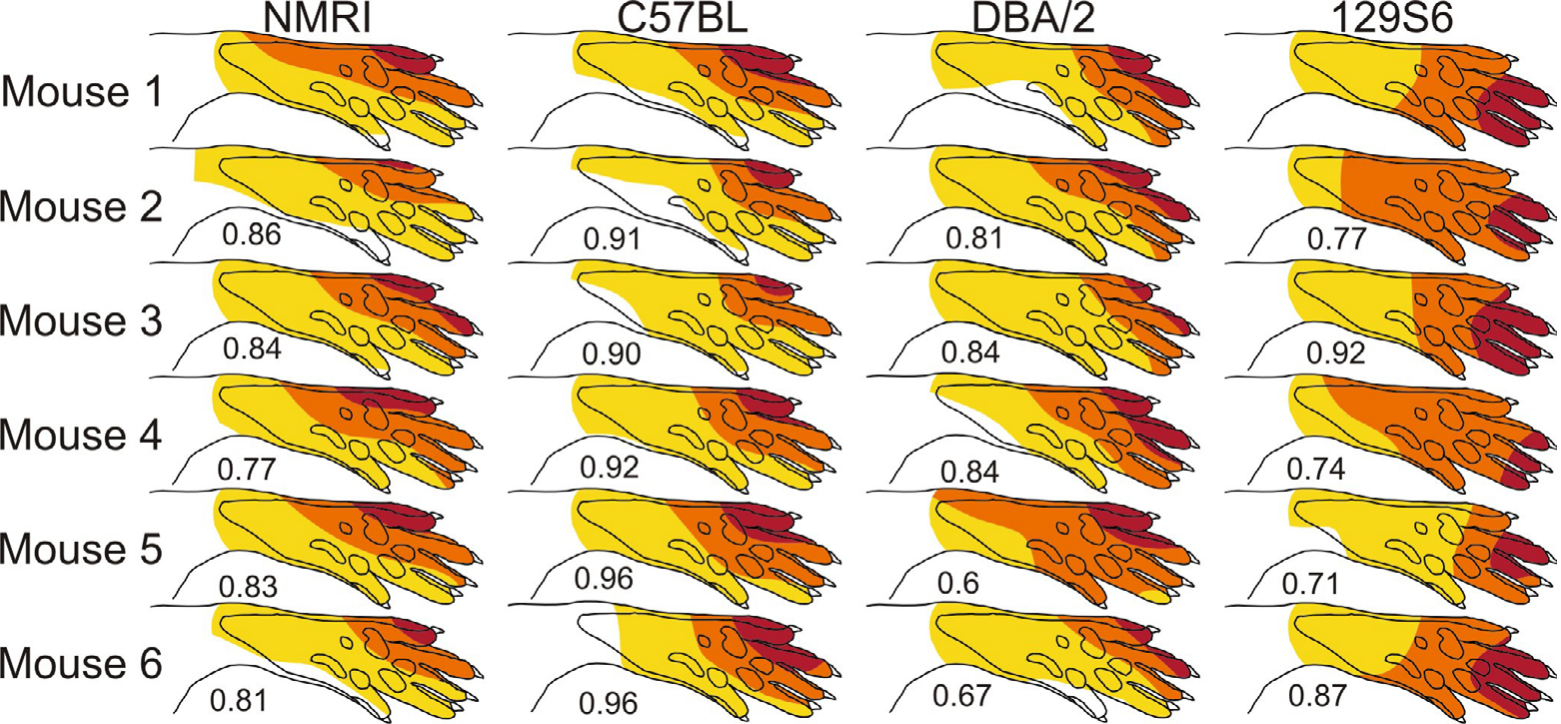
**Comparison of receptive fields in individual mice of the same strain for m. peroneus longus.** Each map is based on data from five consecutive mappings. The Spearman correlation ranged between r = 0.60–0.96 for the strains examined. These correlations were statistically significant for all the muscles studied (range of P values < 0.05 – 0.001).

To assess if mice strains with deficient receptive field organisations exhibit a more variable signal transmission (which could impair the receptive field mapping), we compared the variation in response amplitude of respective foci between different mice strains. No significant difference in variation (i.e. SD) or relative variation (i.e. SD divided by the actual response amplitude) of focus response amplitude was found between different strains (variation were: ± 52, ± 75, ± 39 and ± 56 for NMRI, C57Bl/6, DBA/2 and 129S6/SvEvTac, respectively and relative variation were 0.44, 0.68, 0.64, 0.43 for NMRI, C57Bl/6, DBA/2 and 129S6/SvEvTac, respectively. In addition, it was found that the spatial organisation of the receptive field was very similar for all animals in each strain (Fig. 4). Taken together it appears that the deficient somatosensory imprinting found in the LTP deficient mice strains was not due to an increased variability in signal transmission.

## Discussion

In this study, we have shown that spinal withdrawal reflexes are organised in the same way in NMRI and C57BL/6 mice strains as in rats indicating that a modular organisation is present also in the mice. Interestingly, the two mouse strains with defects in spatial learning [9,10,19,20] and hippocampal LTP [10,13] exhibited more or less impaired somatosensory imprinting in the spinal nociceptive reflex circuits. The defect sensorimotor transformation in these reflex networks often results in mal-directed movements. The implications of these findings for the understanding of the mechanisms underlying functional adaptation of the spinal nociceptive reflexes are discussed below.

A modular organisation of the NWR has previously been described in rats [16], cats [21] and humans [22]. Each reflex module controls a single muscle and has a receptive field whose sensitivity distribution is adapted to the withdrawal pattern in a standing position on contraction in the muscle. Differences in receptive fields between the species mentioned have been found to correspond to anatomical differences. For example, the cat stands on the plantar skin of the digits while the rat press down the entire plantar surface in the standing position, resulting in a difference in withdrawal efficacy of the digit extensor muscles. This difference in movement pattern corresponds to the differences in their receptive fields [21]. Since the hind limb anatomy is very similar in mice and rats, the similarity in receptive fields between the two species indicates that sensory encoding in mouse NWRs is also organised in a motor frame of reference and that the modular organisation is a general principle in mammals.

Two of the mouse strains (129S6/SvEvTac, DBA/2) tested in this study have known defects in their hippocampal LTP (but not LTD) and perform poorly in various spatial learning tasks [10,11,23]. The present study shows that these strains have an abnormal spatial organisation of sensitivity within the NWRs. By contrast, these strains do not differ significantly with respect to reflex thresholds or onset C-fibre latencies within their respective receptive field foci as compared to normal mouse strains. Thus, mechanisms determining spatial distribution of sensitivity and general reflex sensitivity must be at least partly different. Importantly, it therefore appears that somatosensory imprinting only results in relative differences in connection strength in the reflex circuits.

The deficient mechanisms causing lack of LTP in the 129S6/SvEvTac and DBA/2 appear to be at least partly different. The 129S6/SvEvTac mice strain has been reported to have deficient NMDA receptors and deficient LTP induction [10,24]. DBA/2, on the other hand, has a normal induction of LTP but their ability to maintain the LTP is impaired. It is thus conceivable that the 129S6/SvEvTac and DBA/2 mice fail to functionally adapt the NWRs due to deficient NMDA receptor mechanisms and deficient consolidation mechanisms, respectively. It is well known that NMDA receptors are important for fine tuning the spinal cord during development [25]. In fact, the tactile somatotopic organisation and the NWR receptive fields are distorted by the NMDA antagonists MK801 if administered topically on the spinal cord the first two to three weeks after birth [26]. It is thus possible that NMDA dependent learning mechanisms are involved in somatosensory imprinting.

## Conclusion

The present study was undertaken partly with the aim to provide a basis for further studies of nociceptive processing and somatosensory imprinting in the mice. Whereas the mice strains NMRI and C57BL stands out as good choices for such studies, the 129S6/SvEvTac and DBA/2 mice strains, on the other hand, may not be suitable. One important implication of the present results is therefore that it is necessary to pay attention to the wild-type mouse strain used in studies of pain related mechanisms. Interestingly, the present data also indicate that the mice strains studied with deficient LTP also exhibit poor adaptation of the NWRs, suggesting a role of LTP like mechanisms in somatosensory imprinting.

## Authors' contributions

JT participated in designing the study, carried out experimental studies and in drafting the manuscript. JS conceived of the study, and participated in its design and in drafting the manuscript. Both authors read and approved the final manuscript.
